# Supplementary material for: Development and validation of the person-centered postnatal care scale for low- and middle-income countries
Source: Reprod Health. 2026 Apr 11;23:105. doi: 10.1186/s12978-026-02330-z (PMC13200364; doi:10.1186/s12978-026-02330-z)
Supplement: Supplementary file 1 — Supplementary Material 1: Appendix 1: Characteristic of the initial validation study population, Ghana,N=267. Appendix 2: Distribution of all Initial PCPNC items, Ghana only, N=268. Appendix 3: Distribution of revised PCPNC questions included in CPIPE Baseline Sample for Ghana and Kenya, N=1,394. Appendix 4: Exploratory Factor analysis of 54 PCPNC items, CPIPE Baseline Sample for Ghana and Kenya, N=1,376. Appendix 5: Exploratory factor analysis by country of 38 retained items, CPIPE Baseline Sample for Ghana and Kenya. [file 12978_2026_2330_MOESM1_ESM.docx]

| **Appendix 1: Characteristic of the initial validation study population, Ghana, N=267** | | |
| --- | --- | --- |
|  | No. | % |
| Age |  |  |
| Below 20 yrs | 18 | 6.7 |
| 20-24 | 89 | 33.2 |
| 25-29 | 73 | 27.2 |
| 30-34 | 64 | 23.9 |
| 35-39 | 18 | 6.7 |
| 40 or more | 6 | 2.2 |
|  |  |  |
| Marital status |  |  |
| Single | 17 | 6.3 |
| Married/Partnered | 251 | 93.7 |
|  |  |  |
| Number of times given birth in total | |  |
| None | 1 | 0.4 |
| One | 89 | 33.2 |
| Two | 78 | 29.1 |
| Three | 52 | 19.4 |
| Four | 28 | 10.4 |
| 5 or more | 20 | 7.5 |
|  |  |  |
| Months of postpartum |  |  |
| 3 wks or less | 39 | 14.6 |
| 4-7 wks | 57 | 21.3 |
| 8-11 wks | 35 | 13.1 |
| 3 months | 53 | 19.8 |
| 4 months | 36 | 13.4 |
| 5 months | 28 | 10.4 |
| 6 months | 20 | 7.5 |
|  |  |  |
| Highest grade completed at school | |  |
| None | 13 | 4.9 |
| Primary or less | 51 | 19 |
| Post-primary/vocational | 90 | 33.6 |
| Secondary | 87 | 32.5 |
| College/University | 27 | 10.1 |
|  |  |  |
| Partner's highest grade completed at school | | |
| None | 19 | 7.1 |
| Primary or less | 41 | 15.3 |
| Post-primary/vocational | 56 | 20.9 |
| Secondary | 79 | 29.5 |
| College/University | 58 | 21.6 |
| Refused to answer | 15 | 5.6 |
|  |  |  |
| Occupation |  |  |
| Farming | 46 | 17.2 |
| Trading/selling | 52 | 19.4 |
| Hairdressing/dressmaking/Craftsmanship | 59 | 22 |
| Housewife/unemployed | 68 | 25.4 |
| Teacher/Student | 19 | 7.1 |
| Other | 24 | 9 |
|  |  |  |
| Partner's occupation |  |  |
| Farming | 78 | 29.1 |
| Trading/selling | 30 | 11.2 |
| Hairdressing/dressmaking/Craftsmanship | 28 | 10.4 |
| unemployed | 12 | 4.5 |
| Teacher/Student | 40 | 14.9 |
| Motor/Driver/Mechanic | 15 | 5.6 |
| Other | 65 | 24.3 |
|  |  |  |
| Read and write |  |  |
| No, cannot read and write | 69 | 25.7 |
| Yes, but with some difficulty with reading or writing | 90 | 33.6 |
| Yes, can read and write very well | 109 | 40.7 |
|  |  |  |
| Money recieved in a month (GH cedis) | |  |
| None/Undisclosed | 28 | 10.4 |
| 500 or less | 211 | 78.8 |
| More than 500 | 29 | 10.8 |
|  |  |  |
| Household wealth quintile |  |  |
| First | 53 | 19.8 |
| Second | 62 | 23.1 |
| Third | 65 | 24.3 |
| Fourth | 42 | 15.7 |
| Fifth | 46 | 17.2 |
|  |  |  |
| Religion |  |  |
| Christian | 243 | 90.7 |
| Muslim | 19 | 7.1 |
| Traditionalist | 6 | 2.2 |
|  |  |  |
| Ethnicity |  |  |
| Kasem | 104 | 38.8 |
| Nankani/Frafra | 123 | 45.9 |
| Builsa | 31 | 11.6 |
| Other | 10 | 3.8 |
|  |  |  |
| ANC frequency |  |  |
| No | 1 | 0.4 |
| Yes | 267 | 99.6 |
| 2 or less | 2 | 0.7 |
| 3 to 5 months | 37 | 13.9 |
| 6 to 8 months | 92 | 34.5 |
| 9 or more times | 136 | 50.9 |
|  |  |  |
| Gestational age at birth |  |  |
| Less than 9 months | 15 | 5.6 |
| 9 months | 224 | 83.6 |
| 10 months | 29 | 10.8 |
|  |  |  |
| Birth location |  |  |
| Govt. Hospital | 119 | 44.4 |
| Health Center/other lower level gov't facility | 105 | 39.2 |
| Mission/Private Hospital | 41 | 15.3 |
| Home/TBA | 3 | 1.1 |
|  |  |  |
| Reason for first postnatal care | |  |
| For routine checkup | 251 | 93.7 |
| Because of a problem | 16 | 6 |
| Dont Know/NA | 1 | 0.4 |
|  |  |  |
| Number of postnatal care visits | |  |
| Once | 57 | 21.3 |
| Two times | 54 | 20.1 |
| 3 times | 66 | 24.6 |
| 4 or more | 91 | 34 |

| **Appendix 2: Distribution of all PCPNC items, Ghana only, N=268** | | |
| --- | --- | --- |
|  | No. | % |
| How did you feel about the amount of time it took you to retrieve your folder? | | |
| It was just right | 154 | 57.5 |
| It was somewhat long | 42 | 15.7 |
| It was very long | 5 | 1.9 |
| It was extremely long | 1 | 0.4 |
| Not applicable/ home visit | 66 | 24.6 |
|  |  |  |
| How did you feel about the amount of time it took you to retrieve your baby/ies? | | |
| It was just right | 98 | 36.6 |
| It was somewhat long | 22 | 8.2 |
| It was very long | 3 | 1.1 |
| Not applicable | 145 | 54.1 |
|  |  |  |
| How did you feel about the amount of time you had to wait for you and your baby/ies? | | |
| It was just right | 188 | 70.1 |
| It was somewhat long | 65 | 24.3 |
| It was very long | 8 | 3 |
| It was extremely long | 1 | 0.4 |
| Not applicable/home visit | 6 | 2.2 |
|  |  |  |
| How did you feel about the amount of time you waited to get your labs done ? | | |
| It was just right | 98 | 36.6 |
| It was somewhat long | 42 | 15.7 |
| It was very long | 6 | 2.2 |
| It was extremely long | 4 | 1.5 |
| Not applicable/ home visit | 118 | 44 |
|  |  |  |
| How did you feel about the amount of time you waited to get your baby/iess labs ? | | |
| It was just right | 90 | 33.6 |
| It was somewhat long | 42 | 15.7 |
| It was very long | 7 | 2.6 |
| It was extremely long | 2 | 0.7 |
| Not applicable | 127 | 47.4 |
|  |  |  |
| How did you feel about the amount of time you waited to get your drugs? | | |
| It was just right | 120 | 44.8 |
| It was somewhat long | 26 | 9.7 |
| It was very long | 4 | 1.5 |
| Not applicable/ home visit | 118 | 44 |
|  |  |  |
| How did you feel about the amount of time you waited to get your baby/iess drugs? | | |
| It was just right | 155 | 57.8 |
| It was somewhat long | 30 | 11.2 |
| It was very long | 5 | 1.9 |
| It was extremely long | 1 | 0.4 |
| Not applicable/ home visit | 77 | 28.7 |
|  |  |  |
| How did you feel about the amount of time the doctor spent with you? | | |
| It was just right | 243 | 90.7 |
| It was too long | 7 | 2.6 |
| It was somewhat short | 17 | 6.3 |
| It was very short | 1 | 0.4 |
|  |  |  |
| How did you feel about the amount of time the doctor spent with your baby/ies? | | |
| It was just right | 244 | 91 |
| It was too long | 6 | 2.2 |
| It was somewhat short | 16 | 6 |
| It was very short | 2 | 0.7 |
|  |  |  |
| Did you like how you were received when you arrived at the health facility? | | |
| Yes, a few times | 8 | 3 |
| Yes, most of the time | 53 | 19.8 |
| Yes, all the time | 207 | 77.2 |
|  |  |  |
| Were your family members allowed to accompany you and your baby if you wished? | | |
| No, never | 1 | 0.4 |
| Yes, a few times | 16 | 6 |
| Yes, most of the time | 22 | 8.2 |
| Yes, all the time | 142 | 53 |
| Not applicable/I did not visit with any company | 87 | 32.5 |
|  |  |  |
| Did the health workers introduce themselves to you when they first saw you? | | |
| No, none of them | 134 | 50 |
| Yes, a few of them | 25 | 9.3 |
| Yes, most of them | 31 | 11.6 |
| Yes, all of them | 78 | 29.1 |
|  |  |  |
| Did they call you by your name (or appropriately)? | | |
| No, never | 40 | 14.9 |
| Yes, a few times | 32 | 11.9 |
| Yes, most of the time | 25 | 9.3 |
| Yes, all the time | 170 | 63.4 |
| Refused to answer | 1 | 0.4 |
|  |  |  |
| Did they call your baby/ies by their name (or appropriately)? | | |
| No, never | 104 | 38.8 |
| Yes, a few times | 19 | 7.1 |
| Yes, most of the time | 28 | 10.4 |
| Yes, all the time | 116 | 43.3 |
| Refused to answer | 1 | 0.4 |
|  |  |  |
| Did they treat you with respect? | |  |
| Yes, a few times | 3 | 1.1 |
| Yes, most of the time | 24 | 9 |
| Yes, all the time | 241 | 89.9 |
|  |  |  |
| Did they treat your baby/ies with respect? | | |
| Yes, a few times | 4 | 1.5 |
| Yes, most of the time | 19 | 7.1 |
| Yes, all the time | 245 | 91.4 |
|  |  |  |
| Did the health workers respect your family or companions who were with you? | | |
| No, never | 1 | 0.4 |
| Yes, a few times | 4 | 1.5 |
| Yes, most of the time | 22 | 8.2 |
| Yes, all the time | 138 | 51.5 |
| Not applicable/I did not have family or companions present | 103 | 38.4 |
|  |  |  |
| Did the health workers involve you in decisions about your care? | | |
| No, never | 19 | 7.1 |
| Yes, a few times | 5 | 1.9 |
| Yes, most of the time | 43 | 16 |
| Yes, all the time | 201 | 75 |
|  |  |  |
| Did the health workers involve you in decisions about your baby/iess care? | | |
| No, never | 20 | 7.5 |
| Yes, a few times | 2 | 0.7 |
| Yes, most of the time | 39 | 14.6 |
| Yes, all the time | 207 | 77.2 |
|  |  |  |
| Did you feel health workers listened to you? | | |
| Yes, a few times | 10 | 3.7 |
| Yes, most of the time | 34 | 12.7 |
| Yes, all the time | 224 | 83.6 |
|  |  |  |
| Did the health care provider consider your beliefs and values in deciding your care? | | |
| No, never | 54 | 20.1 |
| Yes, a few times | 7 | 2.6 |
| Yes, most of the time | 44 | 16.4 |
| Yes, all the time | 158 | 59 |
| Dont know | 5 | 1.9 |
|  |  |  |
| Did you feel your knowledge was valued? | |  |
| No, never | 5 | 1.9 |
| Yes, a few times | 9 | 3.4 |
| Yes, most of the time | 47 | 17.5 |
| Yes, all the time | 204 | 76.1 |
| Dont know | 3 | 1.1 |
|  |  |  |
| Did the health care provider respect your decisions you took alone in the absence of your partner? | | |
| No, never | 24 | 9 |
| Yes, a few times | 7 | 2.6 |
| Yes, most of the time | 56 | 20.9 |
| Yes, all the time | 139 | 51.9 |
| Not applicable/I did not have a partner | 38 | 14.2 |
| Refused to answer | 4 | 1.5 |
|  |  |  |
| Did they explain to you why they were doing examinations or procedures on you? | | |
| No, never | 12 | 4.5 |
| Yes, a few times | 7 | 2.6 |
| Yes, most of the time | 40 | 14.9 |
| Yes, all the time | 209 | 78 |
|  |  |  |
| Did they explain to you why they were doing examinations or procedures on your baby? | | |
| No, never | 13 | 4.9 |
| Yes, a few times | 10 | 3.7 |
| Yes, most of the time | 34 | 12.7 |
| Yes, all the time | 211 | 78.7 |
|  |  |  |
| Did you feel you understood the purpose of any tests or procedures you were asked to do for your baby? | | |
| No, never | 10 | 3.7 |
| Yes, a few times | 4 | 1.5 |
| Yes, most of the time | 31 | 11.6 |
| Yes, all the time | 185 | 69 |
| Not applicable | 38 | 14.2 |
|  |  |  |
| Did you feel you understood the purpose of any tests or procedures you were asked to do? | | |
| No, never | 16 | 6 |
| Yes, a few times | 3 | 1.1 |
| Yes, most of the time | 40 | 14.9 |
| Yes, all the time | 208 | 77.6 |
| Refused to answer | 1 | 0.4 |
|  |  |  |
| Did they explain to you why they were giving or prescribing you any medicine? | | |
| No, never | 7 | 2.6 |
| Yes, a few times | 4 | 1.5 |
| Yes, most of the time | 25 | 9.3 |
| Yes, all the time | 135 | 50.4 |
| Not applicable/I was not given any medicine | 97 | 36.2 |
|  |  |  |
| Did they explain to you why they were giving or prescribing your baby/ies any medicine? | | |
| No, never | 17 | 6.3 |
| Yes, a few times | 5 | 1.9 |
| Yes, most of the time | 40 | 14.9 |
| Yes, all the time | 206 | 76.9 |
|  |  |  |
| Did you feel you understood the purpose of any medicines you were given or presceibed? | | |
| No, never | 8 | 3 |
| Yes, a few times | 6 | 2.2 |
| Yes, most of the time | 23 | 8.6 |
| Yes, all the time | 147 | 54.9 |
| Not applicable | 84 | 31.3 |
|  |  |  |
| Did you feel you understood the purpose of any medicines you were given or prescribed? | | |
| No, never | 15 | 5.6 |
| Yes, a few times | 8 | 3 |
| Yes, most of the time | 38 | 14.2 |
| Yes, all the time | 205 | 76.5 |
| Refused to answer | 2 | 0.7 |
|  |  |  |
| Did you feel you could ask the health workers any questions you had about yourself? | | |
| No, never | 6 | 2.2 |
| Yes, a few times | 7 | 2.6 |
| Yes, most of the time | 28 | 10.4 |
| Yes, all the time | 227 | 84.7 |
|  |  |  |
| Did you feel you could ask the health workers any questions you had about your baby? | | |
| No, never | 4 | 1.5 |
| Yes, a few times | 6 | 2.2 |
| Yes, most of the time | 27 | 10.1 |
| Yes, all the time | 231 | 86.2 |
|  |  |  |
| Did you hold back from asking questions about yourself for any reason? | | |
| No, never | 227 | 84.7 |
| Yes, a few times | 4 | 1.5 |
| Yes, most of the time | 1 | 0.4 |
| Yes, all the time | 1 | 0.4 |
| Not applicable/I did not have any questions | 35 | 13.1 |
|  |  |  |
| Did you hold back from asking questions about your baby/ies for any reason? | | |
| No, never | 261 | 97.4 |
| Yes, a few times | 3 | 1.1 |
| Yes, most of the time | 2 | 0.7 |
| Yes, all the time | 2 | 0.7 |
|  |  |  |
| Did the health workers at the facility ask you if you had any questions about yourself? | | |
| No, never | 43 | 16 |
| Yes, a few times | 18 | 6.7 |
| Yes, most of the time | 47 | 17.5 |
| Yes, all the time | 160 | 59.7 |
|  |  |  |
| Did the health workers at the facility ask you if you had any questions about yoour baby? | | |
| No, never | 38 | 14.2 |
| Yes, a few times | 8 | 3 |
| Yes, most of the time | 41 | 15.3 |
| Yes, all the time | 181 | 67.5 |
|  |  |  |
| Did they encourage you to ask questions about yourself? | | |
| No, never | 37 | 13.8 |
| Yes, a few times | 27 | 10.1 |
| Yes, most of the time | 49 | 18.3 |
| Yes, all the time | 154 | 57.5 |
| Refused to answer | 1 | 0.4 |
|  |  |  |
| Did they encourage you to ask questions about your baby/ies? | | |
| No, never | 36 | 13.4 |
| Yes, a few times | 18 | 6.7 |
| Yes, most of the time | 44 | 16.4 |
| Yes, all the time | 170 | 63.4 |
|  |  |  |
| Do you feel your questions about yourself were adequately answered when you asked? | | |
| No, never | 2 | 0.7 |
| Yes, a few times | 6 | 2.2 |
| Yes, most of the time | 29 | 10.8 |
| Yes, all the time | 154 | 57.5 |
| Not applicable/I did not ask any questions | 77 | 28.7 |
|  |  |  |
| Do you feel your questions about your baby/ies were adequately answered when you asked? | | |
| No, never | 1 | 0.4 |
| Yes, a few times | 6 | 2.2 |
| Yes, most of the time | 25 | 9.3 |
| Yes, all the time | 169 | 63.1 |
| Not applicable/I did not ask any questions | 67 | 25 |
|  |  |  |
| Did they check that you understood the information that was given to you about yourself? | | |
| No, never | 21 | 7.8 |
| Yes, a few times | 11 | 4.1 |
| Yes, most of the time | 56 | 20.9 |
| Yes, all the time | 180 | 67.2 |
|  |  |  |
| Did they check that you understood the information that was given to you about your baby? | | |
| No, never | 17 | 6.3 |
| Yes, a few times | 12 | 4.5 |
| Yes, most of the time | 42 | 15.7 |
| Yes, all the time | 197 | 73.5 |
|  |  |  |
| Did the health workers speak to you in a language you could understand? | | |
| Yes, a few of them | 6 | 2.2 |
| Yes, most of them | 26 | 9.7 |
| Yes, all of them | 235 | 87.7 |
| Refused to answer | 1 | 0.4 |
|  |  |  |
| Did they speak to you using words you could understand? | | |
| No, never | 2 | 0.7 |
| Yes, a few times | 5 | 1.9 |
| Yes, most of the time | 42 | 15.7 |
| Yes, all the time | 219 | 81.7 |
|  |  |  |
| Did the health workers ask your permission before examining or doing procedures on you? | | |
| No, never | 3 | 1.1 |
| Yes, a few times | 4 | 1.5 |
| Yes, most of the time | 25 | 9.3 |
| Yes, all the time | 236 | 88.1 |
|  |  |  |
| Did the health workers ask your permission before examining or doing procedures on your baby? | | |
| No, never | 3 | 1.1 |
| Yes, a few times | 3 | 1.1 |
| Yes, most of the time | 21 | 7.9 |
| Yes, all the time | 240 | 89.9 |
|  |  |  |
| Did you feel forced into a decision by health workers? | | |
| No, never | 238 | 88.8 |
| Yes, a few times | 1 | 0.4 |
| Yes, most of the time | 5 | 1.9 |
| Yes, all the time | 24 | 9 |
|  |  |  |
| Did you feel they took the best care of you? | | |
| No, never | 3 | 1.1 |
| Yes, a few times | 4 | 1.5 |
| Yes, most of the time | 41 | 15.3 |
| Yes, all the time | 219 | 81.7 |
| Refused to answer | 1 | 0.4 |
|  |  |  |
| Did you feel they took the best care of your baby/ies? | | |
| No, never | 4 | 1.5 |
| Yes, a few times | 2 | 0.7 |
| Yes, most of the time | 28 | 10.4 |
| Yes, all the time | 234 | 87.3 |
|  |  |  |
| Did they ask you about your physical health? | | |
| No, never | 37 | 13.8 |
| Yes, a few times | 7 | 2.6 |
| Yes, most of the time | 36 | 13.4 |
| Yes, all the time | 185 | 69 |
| Refused to answer | 3 | 1.1 |
|  |  |  |
| Did they ask you about your baby/iess physical health? | | |
| No, never | 26 | 9.7 |
| Yes, a few times | 15 | 5.6 |
| Yes, most of the time | 37 | 13.8 |
| Yes, all the time | 189 | 70.5 |
| Refused to answer | 1 | 0.4 |
|  |  |  |
| Did they ask you about your mental health? | | |
| No, never | 83 | 31 |
| Yes, a few times | 27 | 10.1 |
| Yes, most of the time | 44 | 16.4 |
| Yes, all the time | 114 | 42.5 |
|  |  |  |
| Did they ask you about your mood? | |  |
| No, never | 94 | 35.1 |
| Yes, a few times | 29 | 10.8 |
| Yes, most of the time | 39 | 14.6 |
| Yes, all the time | 106 | 39.6 |
|  |  |  |
| Did they give you the support to deal with your mental and/or emotional health? | | |
| No, never | 21 | 7.8 |
| Yes, a few times | 12 | 4.5 |
| Yes, most of the time | 45 | 16.8 |
| Yes, all the time | 92 | 34.3 |
| Not applicable/I did not need emotional support | 98 | 36.6 |
|  |  |  |
| Did the health care provider meet your other health needs? | | |
| No, never | 16 | 6 |
| Yes, a few times | 20 | 7.5 |
| Yes, most of the time | 64 | 24 |
| Yes, all the time | 167 | 62.5 |
|  |  |  |
| Did the health care provider meet your baby/ies other health needs? | | |
| No, never | 8 | 3 |
| Yes, a few times | 11 | 4.1 |
| Yes, most of the time | 52 | 19.4 |
| Yes, all the time | 197 | 73.5 |
|  |  |  |
| Were you counselled by the health worker/s during your postnatal care? | | |
| No, never | 23 | 8.6 |
| Yes, a few times | 18 | 6.7 |
| Yes, most of the time | 46 | 17.2 |
| Yes, all the time | 180 | 67.4 |
|  |  |  |
| Did the health care worker/s record/write you or your baby/ies information in your card? | | |
| No, never | 12 | 4.5 |
| Yes, a few times | 9 | 3.4 |
| Yes, most of the time | 18 | 6.7 |
| Yes, all the time | 229 | 85.4 |
|  |  |  |
| Did you feel your health information was kept confidential by the health workers? | | |
| No, never | 8 | 3 |
| Yes, a few times | 4 | 1.5 |
| Yes, most of the time | 49 | 18.3 |
| Yes, all the time | 197 | 73.5 |
| Dont know | 10 | 3.7 |
|  |  |  |
| Did you feel you could discuss your problems or your baby/iess problems with the health worker without others overhearing? | | |
| No, never | 29 | 10.8 |
| Yes, a few times | 13 | 4.9 |
| Yes, most of the time | 30 | 11.2 |
| Yes, all the time | 194 | 72.4 |
| Refused to answer | 2 | 0.7 |
|  |  |  |
| Did you feel the health workers avoided, ignored, or neglected you? | | |
| No, never | 264 | 98.5 |
| Yes, a few times | 1 | 0.4 |
| Yes, many times | 3 | 1.1 |
|  |  |  |
| Did you feel the health workers avoided, ignored, or neglected your baby/ies? | | |
| No, never | 267 | 99.6 |
| Yes, a few times | 1 | 0.4 |
|  |  |  |
| Did you feel they talked to you or about you badly (For example, shouted at you,.) | | |
| No, never | 267 | 99.6 |
| Yes, a few times | 1 | 0.4 |
|  |  |  |
| Did you feel they talked to your baby/ies or your baby/ies badly | | |
| No, never | 268 | 100 |
|  |  |  |
| Did you feel they handled you badly (For example pushed, beat, slapped, pinched,) | | |
| No, never | 266 | 99.6 |
| Refused to answer | 1 | 0.4 |
|  |  |  |
| Did you feel they handled your baby/ies badly (For example pushed, beat, slapped) | | |
| No, never | 266 | 99.3 |
| Yes, once | 1 | 0.4 |
| Yes, a few times | 1 | 0.4 |
|  |  |  |
| Did you feel that the health care providers recognized and responded if you were in pain? | | |
| No, never | 18 | 6.7 |
| Yes, a few times | 22 | 8.2 |
| Yes, most of the time | 46 | 17.2 |
| Yes, all the time | 126 | 47 |
| Not applicable | 56 | 20.9 |
|  |  |  |
| Did you feel that the health care providers recognized and responded if your baby was in pain? | | |
| No, never | 18 | 6.7 |
| Yes, a few times | 9 | 3.4 |
| Yes, most of the time | 51 | 19 |
| Yes, all the time | 154 | 57.5 |
| Not applicable | 36 | 13.4 |
|  |  |  |
| Did you feel your baby/iess health information was kept confidential by the health provider? | | |
| No, never | 8 | 3 |
| Yes, a few times | 3 | 1.1 |
| Yes, most of the time | 43 | 16 |
| Yes, all the time | 209 | 78 |
| Dont know | 5 | 1.9 |
|  |  |  |
| Did you feel that the health workers discriminated against you in any way? | | |
| No, never | 260 | 97 |
| Yes, a few times | 1 | 0.4 |
| Yes, most of the time | 1 | 0.4 |
| Yes, all the time | 6 | 2.2 |
|  |  |  |
| Did you feel that the health workers discriminated against your baby/ies in any way? | | |
| No, never | 262 | 97.8 |
| Yes, a few times | 1 | 0.4 |
| Yes, all the time | 4 | 1.5 |
| Refused to answer | 1 | 0.4 |
|  |  |  |
| Did you feel you could trust the health workers with regards to your care? | | |
| No, never | 2 | 0.7 |
| Yes, a few times | 7 | 2.6 |
| Yes, most of the time | 71 | 26.5 |
| Yes, all the time | 188 | 70.1 |
|  |  |  |
| Did you feel you could trust the health workers with regards to your baby/iess care? | | |
| No, never | 2 | 0.7 |
| Yes, a few times | 8 | 3 |
| Yes, most of the time | 63 | 23.5 |
| Yes, all the time | 193 | 72 |
| Refused to answer | 2 | 0.7 |
|  |  |  |
| Could you use the washrooms in the facility if you needed to? | | |
| No, never | 22 | 8.2 |
| Yes, a few times | 20 | 7.5 |
| Yes, most of the time | 44 | 16.4 |
| Yes, all the time | 167 | 62.3 |
| No washroom available | 15 | 5.6 |
|  |  |  |
| Did you feel the health facility environment, including the washrooms were clean? | | |
| No, never | 15 | 5.6 |
| Yes, a few times | 17 | 6.3 |
| Yes, most of the time | 79 | 29.5 |
| Yes, all the time | 144 | 53.7 |
| No washroom available | 13 | 4.9 |
|  |  |  |
| Did you feel that the clinic (room you were in) was the right temperature for you? | | |
| No, never | 6 | 2.2 |
| Yes, a few times | 8 | 3 |
| Yes, most of the time | 65 | 24.3 |
| Yes, all the time | 189 | 70.5 |
|  |  |  |
| Did you feel that the clinic (room your baby/ies were in) was the right temperature for your baby? | | |
| No, never | 7 | 2.6 |
| Yes, a few times | 14 | 5.2 |
| Yes, most of the time | 62 | 23.1 |
| Yes, all the time | 184 | 68.7 |
| Refused to answer | 1 | 0.4 |
|  |  |  |
| Do you think there were enough health staff in the facility to care for you and your baby? | | |
| No, never | 7 | 2.6 |
| Yes, a few times | 24 | 9 |
| Yes, most of the time | 67 | 25 |
| Yes, all the time | 169 | 63.1 |
| Dont know | 1 | 0.4 |
|  |  |  |
| Did you feel the health workers were good at what they do? | | |
| Yes, a few of them | 16 | 6 |
| Yes, most of them | 62 | 23.1 |
| Yes, all of them | 189 | 70.5 |
| Dont know | 1 | 0.4 |
|  |  |  |
| Did you feel that the clinic had the proper equipment and medications, for your baby? | | |
| No, never | 51 | 19 |
| Yes, a few times | 61 | 22.8 |
| Yes, most of the time | 82 | 30.6 |
| Yes, all the time | 51 | 19 |
| Dont know | 23 | 8.6 |
|  |  |  |
| Did you feel that the clinic had the proper equipment and medications, for your babie's care | | |
| No, never | 44 | 16.4 |
| Yes, a few times | 51 | 19 |
| Yes, most of the time | 79 | 29.5 |
| Yes, all the time | 49 | 18.3 |
| Dont know/ Not applicable | 45 | 16.8 |
|  |  |  |
| Did the clinic have the vaccinations your baby needed? | | |
| No, never | 31 | 11.6 |
| Yes, a few times | 28 | 10.4 |
| Yes, most of the time | 46 | 17.2 |
| Yes, all the time | 158 | 59 |
| Dont know | 4 | 1.5 |
| Refused to answer | 1 | 0.4 |
|  |  |  |
| Was the facility able to meet your need in view of any disabilities you have? | | |
| No | 8 | 3 |
| Yes, Somewhat | 12 | 4.5 |
| Yes, definitely | 55 | 20.5 |
| Dont know | 1 | 0.4 |
| Not applicable/No disability | 191 | 71.3 |
| Refused to answer | 1 | 0.4 |
|  |  |  |
| In general, did you feel that you and your baby/ies were physically safe in the facility? | | |
| No, never | 2 | 0.7 |
| Yes, a few times | 5 | 1.9 |
| Yes, most of the time | 61 | 22.8 |
| Yes, all the time | 200 | 74.6 |
|  |  |  |
| Did you feel that health care workers blamed you for your illness or condition? | | |
| No, never | 124 | 46.3 |
| Yes, a few times | 2 | 0.7 |
| Yes, most of the time | 11 | 4.1 |
| Yes, all the time | 5 | 1.9 |
| Not applicable/No illness | 126 | 47 |
|  |  |  |
| Did you feel that health care workers blamed you for your baby/iess illness or condition? | | |
| No, never | 132 | 49.3 |
| Yes, a few times | 3 | 1.1 |
| Yes, most of the time | 10 | 3.7 |
| Yes, all the time | 5 | 1.9 |
| Not applicable/No illness | 117 | 43.7 |
| Refused to answer | 1 | 0.4 |
|  |  |  |
| Did you feel that the clinic had the specialists needed to take care of your illness> | | |
| No, never | 29 | 10.8 |
| Yes, a few times | 10 | 3.7 |
| Yes, most of the time | 53 | 19.8 |
| Yes, all the time | 40 | 14.9 |
| Not applicable/No illness | 136 | 50.7 |
|  |  |  |
| Did you feel that the clinic had the specialists needed to take care of your babie's illness? | | |
| No, never | 29 | 10.8 |
| Yes, a few times | 9 | 3.4 |
| Yes, most of the time | 56 | 20.9 |
| Yes, all the time | 49 | 18.3 |
| Not applicable/No illness | 124 | 46.3 |
| Refused to answer | 1 | 0.4 |
|  |  |  |
| Did you feel you received sufficient information about follow up care for you ? | | |
| No, never | 9 | 3.4 |
| Yes, a few times | 5 | 1.9 |
| Yes, most of the time | 30 | 11.2 |
| Yes, all the time | 157 | 58.6 |
| Not applicable/No follow up needed | 67 | 25 |
|  |  |  |
| Did you feel you received sufficient information about follow up care for your baby? | | |
| No, never | 8 | 3 |
| Yes, a few times | 5 | 1.9 |
| Yes, most of the time | 29 | 10.8 |
| Yes, all the time | 176 | 65.7 |
| Not applicable/No follow up needed | 50 | 18.7 |
|  |  |  |
| Were you able to go to your preferred place/clinic for postnatal care? | | |
| No, never | 8 | 3 |
| Yes, a few times | 6 | 2.2 |
| Yes, most of the time | 29 | 10.8 |
| Yes, all the time | 171 | 63.8 |
| Not applicable/I did not have a preferred place/clinic | 53 | 19.8 |
| Refused to answer | 1 | 0.4 |
|  |  |  |
| Were you able to see your preferred provider for postnatal care? | | |
| No, never | 6 | 2.2 |
| Yes, a few times | 9 | 3.4 |
| Yes, most of the time | 42 | 15.7 |
| Yes, all the time | 120 | 44.8 |
| Not applicable/I did not have a preferred provider | 91 | 34 |
|  |  |  |
| Did any health worker at the facility ask you or your family for an unofficial payment? | | |
| No, never | 267 | 99.6 |
| Yes, most of the time | 1 | 0.4 |
|  |  |  |
| Did you get postnatal care in the same place every time for you and your baby/ies? | | |
| No, I didnt | 3 | 1.1 |
| Yes, a few times | 16 | 6 |
| Yes, most of the time | 26 | 9.7 |
| Yes, all the time | 223 | 83.2 |

| **Appendix 3: Distribution of revised PCPNC questions included in CPIPE Baseline Sample for Ghana and Kenya, N=1,394** | | | | | | | | |
| --- | --- | --- | --- | --- | --- | --- | --- | --- |
|  | Kenya (N=670) | |  | Ghana (N=724) | |  | Total (N=1,394) | |
|  | No. | % |  | No. | % |  | No. | % |
| How did you feel about the amount of time you had to wait for you and your baby/ | | | | | | | |  |
| It was just right | 448 | 66.9 |  | 469 | 64.8 |  | 917 | 65.8 |
| It was somewhat long | 132 | 19.7 |  | 184 | 25.4 |  | 316 | 22.7 |
| It was very long | 74 | 11 |  | 58 | 8 |  | 132 | 9.5 |
| It was extremely long | 15 | 2.2 |  | 9 | 1.2 |  | 24 | 1.7 |
| Not applicable/home visit | 1 | 0.1 |  | 3 | 0.4 |  | 4 | 0.3 |
| Refused to answer | 0 | 0 |  | 1 | 0.1 |  | 1 | 0.1 |
|  |  |  |  |  |  |  |  |  |
| How did you feel about the amount of time you waited to get labs done or get dru | | | | | | | |  |
| It was just right | 301 | 44.9 |  | 399 | 55.1 |  | 700 | 50.2 |
| It was somewhat long | 104 | 15.5 |  | 114 | 15.7 |  | 218 | 15.6 |
| It was very long | 39 | 5.8 |  | 67 | 9.3 |  | 106 | 7.6 |
| It was extremely long | 5 | 0.7 |  | 15 | 2.1 |  | 20 | 1.4 |
| Not applicable/ home visit | 221 | 33 |  | 128 | 17.7 |  | 349 | 25 |
| Refused to answer | 0 | 0 |  | 1 | 0.1 |  | 1 | 0.1 |
|  |  |  |  |  |  |  |  |  |
| How did you feel about the amount of time the health worker spent with you and y | | | | | | | | |
| It was just right | 615 | 91.8 |  | 638 | 88.1 |  | 1,253.00 | 89.9 |
| It was too long | 35 | 5.2 |  | 47 | 6.5 |  | 82 | 5.9 |
| It was somewhat short | 15 | 2.2 |  | 25 | 3.5 |  | 40 | 2.9 |
| It was very short | 5 | 0.7 |  | 9 | 1.2 |  | 14 | 1 |
| Refused to answer | 0 | 0 |  | 5 | 0.7 |  | 5 | 0.4 |
|  |  |  |  |  |  |  |  |  |
| Did you like how you were received when you arrived at the health facility? | | | | | | | |  |
| No, never | 10 | 1.5 |  | 37 | 5.1 |  | 47 | 3.4 |
| Yes, a few times | 83 | 12.4 |  | 93 | 12.9 |  | 176 | 12.6 |
| Yes, most of the time | 133 | 19.9 |  | 221 | 30.6 |  | 354 | 25.4 |
| Yes, all the time | 436 | 65.1 |  | 368 | 50.9 |  | 804 | 57.7 |
| Not applicable | 8 | 1.2 |  | 4 | 0.6 |  | 12 | 0.9 |
|  |  |  |  |  |  |  |  |  |
| Were your family members allowed to accompany you and your baby if you wished? | | | | | | | | |
| No, never | 5 | 0.7 |  | 1 | 0.1 |  | 6 | 0.4 |
| Yes, a few times | 17 | 2.5 |  | 23 | 3.2 |  | 40 | 2.9 |
| Yes, most of the time | 66 | 9.9 |  | 194 | 26.8 |  | 260 | 18.7 |
| Yes, all the time | 333 | 49.7 |  | 476 | 65.8 |  | 809 | 58.1 |
| Not applicable | 249 | 37.2 |  | 29 | 4 |  | 278 | 20 |
|  |  |  |  |  |  |  |  |  |
| Did the health workers introduce themselves to you when they first saw you? (If | | | | | | | |  |
| No, none of them | 498 | 74.3 |  | 351 | 48.5 |  | 849 | 60.9 |
| Yes, a few of them | 65 | 9.7 |  | 143 | 19.8 |  | 208 | 14.9 |
| Yes, most of them | 39 | 5.8 |  | 134 | 18.5 |  | 173 | 12.4 |
| Yes, all of them | 68 | 10.1 |  | 93 | 12.9 |  | 161 | 11.6 |
| Refused to answer | 0 | 0 |  | 2 | 0.3 |  | 2 | 0.1 |
|  |  |  |  |  |  |  |  |  |
| Did they call you and your baby/ies by your name (or appropriately)? | | | | | | |  |  |
| No, never | 61 | 9.1 |  | 109 | 15.1 |  | 170 | 12.2 |
| Yes, a few times | 48 | 7.2 |  | 104 | 14.4 |  | 152 | 10.9 |
| Yes, most of the time | 119 | 17.8 |  | 217 | 30 |  | 336 | 24.1 |
| Yes, all the time | 442 | 66 |  | 279 | 38.6 |  | 721 | 51.8 |
| N/a-dont know | 0 | 0 |  | 13 | 1.8 |  | 13 | 0.9 |
| Refused to answer | 0 | 0 |  | 1 | 0.1 |  | 1 | 0.1 |
|  |  |  |  |  |  |  |  |  |
| Did they treat you and your baby with respect? | | | |  |  |  |  |  |
| No, never | 2 | 0.3 |  | 7 | 1 |  | 9 | 0.6 |
| Yes, a few times | 30 | 4.5 |  | 26 | 3.6 |  | 56 | 4 |
| Yes, most of the time | 92 | 13.7 |  | 176 | 24.3 |  | 268 | 19.2 |
| Yes, all the time | 545 | 81.3 |  | 514 | 71.1 |  | 1,059.00 | 76 |
| N/a-dont know | 1 | 0.1 |  | 0 | 0 |  | 1 | 0.1 |
|  |  |  |  |  |  |  |  |  |
| Did the health workers respect your family or companions who were with you? | | | | | | | |  |
| No, never | 1 | 0.1 |  | 4 | 0.6 |  | 5 | 0.4 |
| Yes, a few times | 10 | 1.5 |  | 25 | 3.5 |  | 35 | 2.5 |
| Yes, most of the time | 59 | 8.8 |  | 178 | 24.6 |  | 237 | 17 |
| Yes, all the time | 314 | 46.9 |  | 418 | 57.8 |  | 732 | 52.5 |
| N/a-dont know | 284 | 42.4 |  | 98 | 13.6 |  | 382 | 27.4 |
| Refused to answer | 2 | 0.3 |  | 0 | 0 |  | 2 | 0.1 |
|  |  |  |  |  |  |  |  |  |
| Did the health workers involve you in decisions about you and your baby/ies car | | | | | | | |  |
| No, never | 112 | 16.7 |  | 38 | 5.3 |  | 150 | 10.8 |
| Yes, a few times | 85 | 12.7 |  | 102 | 14.1 |  | 187 | 13.4 |
| Yes, most of the time | 144 | 21.5 |  | 195 | 27 |  | 339 | 24.3 |
| Yes, all the time | 313 | 46.7 |  | 386 | 53.4 |  | 699 | 50.2 |
| N/a-dont know | 15 | 2.2 |  | 2 | 0.3 |  | 17 | 1.2 |
| Refused to answer | 1 | 0.1 |  | 0 | 0 |  | 1 | 0.1 |
|  |  |  |  |  |  |  |  |  |
| Did you feel health workers listened to you? | | | |  |  |  |  |  |
| No, never | 11 | 1.6 |  | 11 | 1.5 |  | 22 | 1.6 |
| Yes, a few times | 65 | 9.7 |  | 118 | 16.3 |  | 183 | 13.1 |
| Yes, most of the time | 141 | 21 |  | 202 | 27.9 |  | 343 | 24.6 |
| Yes, all the time | 369 | 55.1 |  | 388 | 53.7 |  | 757 | 54.3 |
| N/a-dont know | 84 | 12.5 |  | 4 | 0.6 |  | 88 | 6.3 |
|  |  |  |  |  |  |  |  |  |
| Did the health care provider consider your beliefs and values in deciding the ca | | | | | | | |  |
| No, never | 89 | 13.3 |  | 173 | 23.9 |  | 262 | 18.8 |
| Yes, a few times | 28 | 4.2 |  | 116 | 16 |  | 144 | 10.3 |
| Yes, most of the time | 50 | 7.5 |  | 140 | 19.4 |  | 190 | 13.6 |
| Yes, all the time | 242 | 36.1 |  | 199 | 27.5 |  | 441 | 31.7 |
| N/a-dont know | 261 | 39 |  | 94 | 13 |  | 355 | 25.5 |
| Refused to answer | 0 | 0 |  | 1 | 0.1 |  | 1 | 0.1 |
|  |  |  |  |  |  |  |  |  |
| Did you feel your knowledge was valued? If need to clarify: Did they appreciate | | | | | | | |  |
| No, never | 35 | 5.2 |  | 46 | 6.4 |  | 81 | 5.8 |
| Yes, a few times | 86 | 12.8 |  | 160 | 22.1 |  | 246 | 17.7 |
| Yes, most of the time | 155 | 23.1 |  | 200 | 27.7 |  | 355 | 25.5 |
| Yes, all the time | 249 | 37.2 |  | 258 | 35.7 |  | 507 | 36.4 |
| N/a-dont know | 144 | 21.5 |  | 59 | 8.2 |  | 203 | 14.6 |
| Refused to answer | 1 | 0.1 |  | 0 | 0 |  | 1 | 0.1 |
|  |  |  |  |  |  |  |  |  |
| Did the health care provider respect your decisions you took alone in the absenc | | | | | | | | |
| No, never | 12 | 1.8 |  | 7 | 1 |  | 19 | 1.4 |
| Yes, a few times | 44 | 6.6 |  | 105 | 14.5 |  | 149 | 10.7 |
| Yes, most of the time | 92 | 13.7 |  | 201 | 27.8 |  | 293 | 21 |
| Yes, all the time | 286 | 42.7 |  | 364 | 50.3 |  | 650 | 46.7 |
| N/a-dont know | 236 | 35.2 |  | 45 | 6.2 |  | 281 | 20.2 |
| Refused to answer | 0 | 0 |  | 1 | 0.1 |  | 1 | 0.1 |
|  |  |  |  |  |  |  |  |  |
| Did they explain to you why they were doing any examinations on you and your bab | | | | | | | | |
| No, never | 129 | 19.3 |  | 81 | 11.2 |  | 210 | 15.1 |
| Yes, a few times | 116 | 17.3 |  | 84 | 11.6 |  | 200 | 14.4 |
| Yes, most of the time | 123 | 18.4 |  | 183 | 25.3 |  | 306 | 22 |
| Yes, all the time | 244 | 36.4 |  | 367 | 50.8 |  | 611 | 43.9 |
| N/a-dont know | 57 | 8.5 |  | 8 | 1.1 |  | 65 | 4.7 |
| Refused to answer | 1 | 0.1 |  | 0 | 0 |  | 1 | 0.1 |
|  |  |  |  |  |  |  |  |  |
| Did you understand the purpose of any tests you were asked to do for yourself an | | | | | | | |  |
| No, never | 80 | 11.9 |  | 39 | 5.4 |  | 119 | 8.5 |
| Yes, a few times | 104 | 15.5 |  | 82 | 11.3 |  | 186 | 13.4 |
| Yes, most of the time | 118 | 17.6 |  | 179 | 24.8 |  | 297 | 21.3 |
| Yes, all the time | 173 | 25.8 |  | 283 | 39.1 |  | 456 | 32.7 |
| N/a-dont know | 194 | 29 |  | 140 | 19.4 |  | 334 | 24 |
| Refused to answer | 1 | 0.1 |  | 0 | 0 |  | 1 | 0.1 |
|  |  |  |  |  |  |  |  |  |
| Did they explain to you why they were giving you any medicines or treatments? | | | | | | | |  |
| No, never | 86 | 12.8 |  | 102 | 14.1 |  | 188 | 13.5 |
| Yes, a few times | 104 | 15.5 |  | 82 | 11.3 |  | 186 | 13.4 |
| Yes, most of the time | 106 | 15.8 |  | 185 | 25.6 |  | 291 | 20.9 |
| Yes, all the time | 215 | 32.1 |  | 330 | 45.6 |  | 545 | 39.1 |
| N/a-dont know | 159 | 23.7 |  | 24 | 3.3 |  | 183 | 13.1 |
|  |  |  |  |  |  |  |  |  |
| Did you you understand the purpose of any medicines, vacines, or treatments give | | | | | | | | |
| No, never | 137 | 20.4 |  | 110 | 15.2 |  | 247 | 17.7 |
| Yes, a few times | 132 | 19.7 |  | 73 | 10.1 |  | 205 | 14.7 |
| Yes, most of the time | 103 | 15.4 |  | 214 | 29.6 |  | 317 | 22.8 |
| Yes, all the time | 247 | 36.9 |  | 309 | 42.7 |  | 556 | 39.9 |
| N/a-dont know | 51 | 7.6 |  | 16 | 2.2 |  | 67 | 4.8 |
| Refused to answer | 0 | 0 |  | 1 | 0.1 |  | 1 | 0.1 |
|  |  |  |  |  |  |  |  |  |
| Did you feel you could ask the health workers any questions you had about yourse | | | | | | | | |
| No, never | 71 | 10.6 |  | 38 | 5.3 |  | 109 | 7.8 |
| Yes, a few times | 105 | 15.7 |  | 125 | 17.3 |  | 230 | 16.5 |
| Yes, most of the time | 108 | 16.1 |  | 190 | 26.3 |  | 298 | 21.4 |
| Yes, all the time | 353 | 52.7 |  | 367 | 50.8 |  | 720 | 51.7 |
| N/a-dont know | 33 | 4.9 |  | 3 | 0.4 |  | 36 | 2.6 |
|  |  |  |  |  |  |  |  |  |
| Did they encourage you to ask questions about yourself? | | | | | |  |  |  |
| No, never | 273 | 40.7 |  | 115 | 15.9 |  | 388 | 27.9 |
| Yes, a few times | 86 | 12.8 |  | 133 | 18.4 |  | 219 | 15.7 |
| Yes, most of the time | 75 | 11.2 |  | 201 | 27.8 |  | 276 | 19.8 |
| Yes, all the time | 229 | 34.2 |  | 274 | 37.9 |  | 503 | 36.1 |
| N/a-dont know | 7 | 1 |  | 0 | 0 |  | 7 | 0.5 |
|  |  |  |  |  |  |  |  |  |
| Do you feel your questions were adequately answered when you asked them? | | | | | | | |  |
| No, never | 36 | 5.4 |  | 51 | 7.1 |  | 87 | 6.2 |
| Yes, a few times | 90 | 13.4 |  | 132 | 18.3 |  | 222 | 15.9 |
| Yes, most of the time | 130 | 19.4 |  | 192 | 26.6 |  | 322 | 23.1 |
| Yes, all the time | 247 | 36.9 |  | 326 | 45.1 |  | 573 | 41.1 |
| N/a-dont know | 166 | 24.8 |  | 22 | 3 |  | 188 | 13.5 |
| Refused to answer | 1 | 0.1 |  | 0 | 0 |  | 1 | 0.1 |
|  |  |  |  |  |  |  |  |  |
| Did they check that you understood the information that was given to you? | | | | | | | |  |
| No, never | 215 | 32.1 |  | 89 | 12.3 |  | 304 | 21.8 |
| Yes, a few times | 91 | 13.6 |  | 168 | 23.2 |  | 259 | 18.6 |
| Yes, most of the time | 101 | 15.1 |  | 177 | 24.5 |  | 278 | 20 |
| Yes, all the time | 179 | 26.7 |  | 277 | 38.3 |  | 456 | 32.7 |
| N/a-dont know | 83 | 12.4 |  | 12 | 1.7 |  | 95 | 6.8 |
| Refused to answer | 1 | 0.1 |  | 0 | 0 |  | 1 | 0.1 |
|  |  |  |  |  |  |  |  |  |
| Did the health workers speak to you in a language you could understand or using | | | | | | | |  |
| No, none of them | 2 | 0.3 |  | 2 | 0.3 |  | 4 | 0.3 |
| Yes, a few of them | 9 | 1.3 |  | 23 | 3.2 |  | 32 | 2.3 |
| Yes, most of them | 52 | 7.8 |  | 164 | 22.7 |  | 216 | 15.5 |
| Yes, all of them | 607 | 90.6 |  | 534 | 73.9 |  | 1,141.00 | 81.9 |
|  |  |  |  |  |  |  |  |  |
| Did the health workers ask your permission before examining or doing procedures | | | | | | | | |
| No, never | 182 | 27.2 |  | 22 | 3 |  | 204 | 14.6 |
| Yes, a few times | 120 | 17.9 |  | 66 | 9.1 |  | 186 | 13.4 |
| Yes, most of the time | 93 | 13.9 |  | 184 | 25.4 |  | 277 | 19.9 |
| Yes, all the time | 254 | 37.9 |  | 448 | 62 |  | 702 | 50.4 |
| N/a-dont know | 21 | 3.1 |  | 3 | 0.4 |  | 24 | 1.7 |
|  |  |  |  |  |  |  |  |  |
| Did you feel forced into a decision by health workers? | | | | |  |  |  |  |
| No, never | 629 | 93.9 |  | 662 | 91.6 |  | 1,291.00 | 92.7 |
| Yes, a few times | 12 | 1.8 |  | 8 | 1.1 |  | 20 | 1.4 |
| Yes, most of the time | 10 | 1.5 |  | 15 | 2.1 |  | 25 | 1.8 |
| Yes, all the time | 18 | 2.7 |  | 37 | 5.1 |  | 55 | 3.9 |
| N/a-dont know | 1 | 0.1 |  | 1 | 0.1 |  | 2 | 0.1 |
|  |  |  |  |  |  |  |  |  |
| Did you feel they took the best care of you and your baby/ies | | | | | |  |  |  |
| No, never | 2 | 0.3 |  | 8 | 1.1 |  | 10 | 0.7 |
| Yes, a few times | 40 | 6 |  | 57 | 7.9 |  | 97 | 7 |
| Yes, most of the time | 143 | 21.3 |  | 228 | 31.5 |  | 371 | 26.6 |
| Yes, all the time | 484 | 72.2 |  | 427 | 59.1 |  | 911 | 65.4 |
| N/a-dont know | 1 | 0.1 |  | 2 | 0.3 |  | 3 | 0.2 |
| Refused to answer | 0 | 0 |  | 1 | 0.1 |  | 1 | 0.1 |
|  |  |  |  |  |  |  |  |  |
| Did they ask you about your physical health? [Local translation:Did they ask yo | | | | | | | |  |
| No, never | 199 | 29.7 |  | 96 | 13.3 |  | 295 | 21.2 |
| Yes, a few times | 75 | 11.2 |  | 73 | 10.1 |  | 148 | 10.6 |
| Yes, most of the time | 110 | 16.4 |  | 192 | 26.6 |  | 302 | 21.7 |
| Yes, all the time | 285 | 42.5 |  | 359 | 49.7 |  | 644 | 46.2 |
| N/a-dont know | 1 | 0.1 |  | 3 | 0.4 |  | 4 | 0.3 |
|  |  |  |  |  |  |  |  |  |
| Did they ask you about your baby/iess physical health? [Local translation:Di | | | | | | | |  |
| No, never | 112 | 16.7 |  | 36 | 5 |  | 148 | 10.6 |
| Yes, a few times | 69 | 10.3 |  | 53 | 7.3 |  | 122 | 8.8 |
| Yes, most of the time | 111 | 16.6 |  | 175 | 24.2 |  | 286 | 20.5 |
| Yes, all the time | 376 | 56.1 |  | 458 | 63.3 |  | 834 | 59.9 |
| N/a-dont know | 2 | 0.3 |  | 1 | 0.1 |  | 3 | 0.2 |
|  |  |  |  |  |  |  |  |  |
| Did they ask you about your mental and emotional health? [Local translation:D | | | | | | | |  |
| No, never | 452 | 67.5 |  | 339 | 46.9 |  | 791 | 56.8 |
| Yes, a few times | 83 | 12.4 |  | 126 | 17.4 |  | 209 | 15 |
| Yes, most of the time | 37 | 5.5 |  | 94 | 13 |  | 131 | 9.4 |
| Yes, all the time | 89 | 13.3 |  | 154 | 21.3 |  | 243 | 17.4 |
| N/a-dont know | 9 | 1.3 |  | 10 | 1.4 |  | 19 | 1.4 |
|  |  |  |  |  |  |  |  |  |
| Did they give you the support to deal with your mental and/or emotional health? | | | | | | | |  |
| No, never | 344 | 51.3 |  | 304 | 42 |  | 648 | 46.5 |
| Yes, a few times | 69 | 10.3 |  | 101 | 14 |  | 170 | 12.2 |
| Yes, most of the time | 27 | 4 |  | 97 | 13.4 |  | 124 | 8.9 |
| Yes, all the time | 67 | 10 |  | 137 | 18.9 |  | 204 | 14.6 |
| N/a-dont know | 163 | 24.3 |  | 84 | 11.6 |  | 247 | 17.7 |
|  |  |  |  |  |  |  |  |  |
| Did the health care provider meet your other health needs?If need to clarify: Ot | | | | | | | |  |
| No, never | 117 | 17.5 |  | 219 | 30.3 |  | 336 | 24.1 |
| Yes, a few times | 93 | 13.9 |  | 98 | 13.6 |  | 191 | 13.7 |
| Yes, most of the time | 107 | 16 |  | 187 | 25.9 |  | 294 | 21.1 |
| Yes, all the time | 157 | 23.4 |  | 205 | 28.4 |  | 362 | 26 |
| N/a-dont know | 196 | 29.3 |  | 14 | 1.9 |  | 210 | 15.1 |
|  |  |  |  |  |  |  |  |  |
| Were you counselled by the health worker/s during your postnatal care? | | | | | | | |  |
| No, never | 398 | 59.4 |  | 113 | 15.6 |  | 511 | 36.7 |
| Yes, a few times | 91 | 13.6 |  | 82 | 11.3 |  | 173 | 12.4 |
| Yes, most of the time | 55 | 8.2 |  | 159 | 22 |  | 214 | 15.4 |
| Yes, all the time | 121 | 18.1 |  | 366 | 50.6 |  | 487 | 35 |
| N/a-dont know | 4 | 0.6 |  | 3 | 0.4 |  | 7 | 0.5 |
| Refused to answer | 1 | 0.1 |  | 0 | 0 |  | 1 | 0.1 |
|  |  |  |  |  |  |  |  |  |
| Did the health care worker/s record/write you or your baby/ies information in yo | | | | | | | |  |
| No, never | 7 | 1 |  | 5 | 0.7 |  | 12 | 0.9 |
| Yes, a few times | 12 | 1.8 |  | 18 | 2.5 |  | 30 | 2.2 |
| Yes, most of the time | 47 | 7 |  | 90 | 12.4 |  | 137 | 9.8 |
| Yes, all the time | 598 | 89.3 |  | 605 | 83.7 |  | 1,203.00 | 86.4 |
| Dont know | 5 | 0.7 |  | 5 | 0.7 |  | 10 | 0.7 |
| Refused to answer | 1 | 0.1 |  | 0 | 0 |  | 1 | 0.1 |
|  |  |  |  |  |  |  |  |  |
| During physical exams (like abdominal and pelvic exams) were you covered up with | | | | | | | | |
| No, never | 7 | 1 |  | 5 | 0.7 |  | 12 | 0.9 |
| Yes, a few times | 14 | 2.1 |  | 63 | 9.1 |  | 77 | 5.6 |
| Yes, most of the time | 128 | 19.1 |  | 183 | 26.4 |  | 311 | 22.8 |
| Yes, all the time | 440 | 65.7 |  | 412 | 59.5 |  | 852 | 62.5 |
| Dont know | 81 | 12.1 |  | 30 | 4.3 |  | 111 | 8.1 |
|  |  |  |  |  |  |  |  |  |
| Did you feel you could discuss your problems or your baby/iess problems with the | | | | | | | | |
| No, never | 149 | 22.2 |  | 47 | 6.5 |  | 196 | 14.1 |
| Yes, a few times | 63 | 9.4 |  | 121 | 16.7 |  | 184 | 13.2 |
| Yes, most of the time | 107 | 16 |  | 175 | 24.2 |  | 282 | 20.2 |
| Yes, all the time | 347 | 51.8 |  | 375 | 51.9 |  | 722 | 51.8 |
| Dont know | 4 | 0.6 |  | 4 | 0.6 |  | 8 | 0.6 |
| Refused to answer | 0 | 0 |  | 1 | 0.1 |  | 1 | 0.1 |
|  |  |  |  |  |  |  |  |  |
| Did you feel the health workers avoided, ignored, or neglected you or your baby? | | | | | | | |  |
| No, never | 660 | 98.5 |  | 712 | 98.5 |  | 1,372.00 | 98.5 |
| Yes, once | 3 | 0.4 |  | 4 | 0.6 |  | 7 | 0.5 |
| Yes, a few times | 6 | 0.9 |  | 5 | 0.7 |  | 11 | 0.8 |
| Yes, many times | 1 | 0.1 |  | 2 | 0.3 |  | 3 | 0.2 |
|  |  |  |  |  |  |  |  |  |
| Did you feel they talked to you or about you or your baby badly (For example, sh | | | | | | | |  |
| No, never | 660 | 98.5 |  | 717 | 99.2 |  | 1,377.00 | 98.9 |
| Yes, once | 5 | 0.7 |  | 6 | 0.8 |  | 11 | 0.8 |
| Yes, a few times | 5 | 0.7 |  | 0 | 0 |  | 5 | 0.4 |
|  |  |  |  |  |  |  |  |  |
| Did you feel they handled you or your baby badly (For example pushed, beat, slap | | | | | | | | |
| No, never | 665 | 99.3 |  | 716 | 99 |  | 1,381.00 | 99.1 |
| Yes, once | 1 | 0.1 |  | 3 | 0.4 |  | 4 | 0.3 |
| Yes, a few times | 3 | 0.4 |  | 1 | 0.1 |  | 4 | 0.3 |
| Yes, many times | 1 | 0.1 |  | 2 | 0.3 |  | 3 | 0.2 |
| Refused to answer | 0 | 0 |  | 1 | 0.1 |  | 1 | 0.1 |
|  |  |  |  |  |  |  |  |  |
| Did you feel that the health care providers recognized and responded if you or y | | | | | | | |  |
| No, never | 29 | 4.3 |  | 24 | 3.3 |  | 53 | 3.8 |
| Yes, a few times | 57 | 8.5 |  | 89 | 12.3 |  | 146 | 10.5 |
| Yes, most of the time | 140 | 20.9 |  | 254 | 35.1 |  | 394 | 28.3 |
| Yes, all the time | 261 | 39 |  | 347 | 48 |  | 608 | 43.6 |
| Not applicable | 183 | 27.3 |  | 9 | 1.2 |  | 192 | 13.8 |
|  |  |  |  |  |  |  |  |  |
| Did you feel you and your baby/iess health information was kept confidential by | | | | | | | |  |
| No, never | 7 | 1 |  | 10 | 1.4 |  | 17 | 1.2 |
| Yes, a few times | 7 | 1 |  | 76 | 10.5 |  | 83 | 6 |
| Yes, most of the time | 127 | 19 |  | 181 | 25 |  | 308 | 22.1 |
| Yes, all the time | 457 | 68.2 |  | 425 | 58.8 |  | 882 | 63.3 |
| Dont know | 72 | 10.7 |  | 31 | 4.3 |  | 103 | 7.4 |
|  |  |  |  |  |  |  |  |  |
| Did you feel that the health workers discriminated against you in any way? | | | | | | | |  |
| No, never | 662 | 98.8 |  | 713 | 98.6 |  | 1,375.00 | 98.7 |
| Yes, a few times | 6 | 0.9 |  | 3 | 0.4 |  | 9 | 0.6 |
| Yes, most of the time | 1 | 0.1 |  | 3 | 0.4 |  | 4 | 0.3 |
| Yes, all the time | 1 | 0.1 |  | 2 | 0.3 |  | 3 | 0.2 |
| Dont know | 0 | 0 |  | 1 | 0.1 |  | 1 | 0.1 |
| Refused to answer | 0 | 0 |  | 1 | 0.1 |  | 1 | 0.1 |
|  |  |  |  |  |  |  |  |  |
| Did you feel you could trust the health workers with regards to you and your bab | | | | | | | |  |
| No, never | 7 | 1 |  | 7 | 1 |  | 14 | 1 |
| Yes, a few times | 31 | 4.6 |  | 78 | 10.8 |  | 109 | 7.8 |
| Yes, most of the time | 186 | 27.8 |  | 262 | 36.2 |  | 448 | 32.2 |
| Yes, all the time | 444 | 66.3 |  | 372 | 51.5 |  | 816 | 58.6 |
| N/a-dont know | 1 | 0.1 |  | 4 | 0.6 |  | 5 | 0.4 |
| Refused to answer | 1 | 0.1 |  | 0 | 0 |  | 1 | 0.1 |
|  |  |  |  |  |  |  |  |  |
| Could you use the washrooms in the facility if you needed to?(If no washroom sel | | | | | | | |  |
| No, never | 28 | 4.2 |  | 91 | 12.6 |  | 119 | 8.5 |
| Yes, a few times | 104 | 15.5 |  | 105 | 14.5 |  | 209 | 15 |
| Yes, most of the time | 88 | 13.1 |  | 149 | 20.6 |  | 237 | 17 |
| Yes, all the time | 411 | 61.3 |  | 358 | 49.5 |  | 769 | 55.2 |
| N/a-dont know | 39 | 5.8 |  | 20 | 2.8 |  | 59 | 4.2 |
|  |  |  |  |  |  |  |  |  |
| Did you feel the health facility environment, including the washrooms were clean | | | | | | | | |
| No, never | 40 | 6 |  | 30 | 4.1 |  | 70 | 5 |
| Yes, a few times | 117 | 17.5 |  | 110 | 15.2 |  | 227 | 16.3 |
| Yes, most of the time | 169 | 25.2 |  | 252 | 34.9 |  | 421 | 30.2 |
| Yes, all the time | 330 | 49.3 |  | 323 | 44.7 |  | 653 | 46.9 |
| N/a-dont know | 14 | 2.1 |  | 8 | 1.1 |  | 22 | 1.6 |
|  |  |  |  |  |  |  |  |  |
| Did you feel that the clinic (the room you and your baby were in) was the right | | | | | | | |  |
| No, never | 14 | 2.1 |  | 42 | 5.8 |  | 56 | 4 |
| Yes, a few times | 47 | 7 |  | 100 | 13.8 |  | 147 | 10.6 |
| Yes, most of the time | 219 | 32.7 |  | 270 | 37.3 |  | 489 | 35.1 |
| Yes, all the time | 371 | 55.4 |  | 303 | 41.9 |  | 674 | 48.4 |
| N/a-dont know | 19 | 2.8 |  | 8 | 1.1 |  | 27 | 1.9 |
|  |  |  |  |  |  |  |  |  |
| Do you think there were enough health staff in the facility to care for you and | | | | | | | |  |
| No, never | 50 | 7.5 |  | 38 | 5.3 |  | 88 | 6.3 |
| Yes, a few times | 61 | 9.1 |  | 58 | 8 |  | 119 | 8.5 |
| Yes, most of the time | 168 | 25.1 |  | 203 | 28.1 |  | 371 | 26.6 |
| Yes, all the time | 390 | 58.2 |  | 422 | 58.4 |  | 812 | 58.3 |
| N/a-dont know | 1 | 0.1 |  | 2 | 0.3 |  | 3 | 0.2 |
|  |  |  |  |  |  |  |  |  |
| Did you feel the health workers were good at what they do? | | | | | |  |  |  |
| No, none of them | 2 | 0.3 |  | 1 | 0.1 |  | 3 | 0.2 |
| Yes, a few of them | 20 | 3 |  | 48 | 6.6 |  | 68 | 4.9 |
| Yes, most of them | 135 | 20.1 |  | 276 | 38.2 |  | 411 | 29.5 |
| Yes, all of them | 513 | 76.6 |  | 395 | 54.6 |  | 908 | 65.2 |
| Dont know | 0 | 0 |  | 3 | 0.4 |  | 3 | 0.2 |
|  |  |  |  |  |  |  |  |  |
| Did you feel that the clinic had the proper equipment and medications, for you a | | | | | | | |  |
| No, never | 25 | 3.7 |  | 55 | 7.6 |  | 80 | 5.7 |
| Yes, a few times | 49 | 7.3 |  | 143 | 19.8 |  | 192 | 13.8 |
| Yes, most of the time | 160 | 23.9 |  | 265 | 36.7 |  | 425 | 30.5 |
| Yes, all the time | 295 | 44 |  | 250 | 34.6 |  | 545 | 39.1 |
| N/a-dont know | 141 | 21 |  | 10 | 1.4 |  | 151 | 10.8 |
|  |  |  |  |  |  |  |  |  |
| In general, did you feel that you and your baby/ies were safe (physically and ps | | | | | | | |  |
| No, never | 4 | 0.6 |  | 10 | 1.4 |  | 14 | 1 |
| Yes, a few times | 27 | 4 |  | 79 | 10.9 |  | 106 | 7.6 |
| Yes, most of the time | 149 | 22.2 |  | 238 | 32.9 |  | 387 | 27.8 |
| Yes, all the time | 488 | 72.8 |  | 393 | 54.4 |  | 881 | 63.2 |
| N/a-dont know | 2 | 0.3 |  | 3 | 0.4 |  | 5 | 0.4 |
|  |  |  |  |  |  |  |  |  |
| Did the clinic have the vaccinations your baby needed? | | | | | |  |  |  |
| No, never | 4 | 0.6 |  | 13 | 1.8 |  | 17 | 1.2 |
| Yes, a few times | 36 | 5.4 |  | 100 | 13.8 |  | 136 | 9.8 |
| Yes, most of the time | 112 | 16.7 |  | 196 | 27.1 |  | 308 | 22.1 |
| Yes, all the time | 510 | 76.1 |  | 407 | 56.3 |  | 917 | 65.8 |
| N/a-dont know | 8 | 1.2 |  | 7 | 1 |  | 15 | 1.1 |
|  |  |  |  |  |  |  |  |  |
| Did you feel that health care workers blamed you for you or your babys illness o | | | | | | | |  |
| No, never | 458 | 68.4 |  | 614 | 84.9 |  | 1,072.00 | 77 |
| Yes, a few times | 11 | 1.6 |  | 14 | 1.9 |  | 25 | 1.8 |
| Yes, most of the time | 5 | 0.7 |  | 18 | 2.5 |  | 23 | 1.7 |
| Yes, all the time | 12 | 1.8 |  | 34 | 4.7 |  | 46 | 3.3 |
| N/a-dont know | 184 | 27.5 |  | 43 | 5.9 |  | 227 | 16.3 |
|  |  |  |  |  |  |  |  |  |
| Did you feel you received sufficient information about follow up care for you an | | | | | | | |  |
| No, never | 78 | 11.6 |  | 55 | 7.6 |  | 133 | 9.5 |
| Yes, a few times | 89 | 13.3 |  | 51 | 7.1 |  | 140 | 10.1 |
| Yes, most of the time | 135 | 20.1 |  | 157 | 21.7 |  | 292 | 21 |
| Yes, all the time | 362 | 54 |  | 458 | 63.3 |  | 820 | 58.9 |
| N/a-dont know | 6 | 0.9 |  | 2 | 0.3 |  | 8 | 0.6 |
|  |  |  |  |  |  |  |  |  |
| Was the facility able to meet your need in view of any disabilities you have? | | | | | | | |  |
| No | 43 | 6.4 |  | 32 | 4.4 |  | 75 | 5.4 |
| Yes, Somewhat | 31 | 4.6 |  | 47 | 6.5 |  | 78 | 5.6 |
| Yes, definitely | 38 | 5.7 |  | 121 | 16.7 |  | 159 | 11.4 |
| Dont know | 3 | 0.4 |  | 1 | 0.1 |  | 4 | 0.3 |
| Not applicable/No disability | 555 | 82.8 |  | 521 | 72.1 |  | 1,076.00 | 77.2 |
| Refused to answer | 0 | 0 |  | 1 | 0.1 |  | 1 | 0.1 |
|  |  |  |  |  |  |  |  |  |
| During your postnatal care, did any health worker at the facility ask you or you | | | | | | | |  |
| No, never | 652 | 97.3 |  | 688 | 95.2 |  | 1,340.00 | 96.2 |
| Yes, a few times | 12 | 1.8 |  | 11 | 1.5 |  | 23 | 1.7 |
| Yes, most of the time | 1 | 0.1 |  | 6 | 0.8 |  | 7 | 0.5 |
| Yes, all the time | 2 | 0.3 |  | 18 | 2.5 |  | 20 | 1.4 |
| Refused to answer | 3 | 0.4 |  | 0 | 0 |  | 3 | 0.2 |

| **Appendix 4: Exploratory Factor analysis of 54 PCPNC items, CPIPE Baseline Sample for Ghana and Kenya, N=1,376** | | | | | | | |
| --- | --- | --- | --- | --- | --- | --- | --- |
|  | **3 factor structure** | | | |  | **single factor structure** | |
| *Subscale/items* | ***Factor1*** | ***Factor2*** | ***Factor3*** | ***Uniqueness*** |  | ***Factor1*** | ***Uniqueness*** |
| Reception | 0.61 |  |  | 0.56 |  | 0.62 | 0.62 |
| Companionship | 0.29 |  |  | 0.71 |  | 0.45 | 0.80 |
| Introductions by provider |  | 0.53 |  | 0.76 |  | 0.32 | 0.89 |
| Called preferred name | 0.46 |  |  | 0.73 |  | 0.43 | 0.81 |
| Treat you with respect | 0.61 |  |  | 0.54 |  | 0.61 | 0.63 |
| Family respected | 0.29 | 0.31 |  | 0.73 |  | 0.51 | 0.74 |
| Involved in decisions | 0.26 | 0.48 |  | 0.56 |  | 0.63 | 0.60 |
| Felt Heard | 0.65 |  |  | 0.41 |  | 0.74 | 0.45 |
| Beliefs valued | 0.45 |  |  | 0.80 |  | 0.37 | 0.87 |
| Knowledge valued | 0.52 | 0.30 |  | 0.51 |  | 0.70 | 0.52 |
| Parental autonomy | 0.49 |  |  | 0.64 |  | 0.53 | 0.72 |
| Explain exams/procedures |  | 0.75 |  | 0.46 |  | 0.58 | 0.67 |
| Understood tests |  | 0.67 |  | 0.53 |  | 0.57 | 0.68 |
| Explain medications |  | 0.76 |  | 0.46 |  | 0.55 | 0.70 |
| Understood medicines |  | 0.79 |  | 0.43 |  | 0.55 | 0.70 |
| Could ask any questions | 0.38 | 0.38 |  | 0.56 |  | 0.65 | 0.57 |
| Encouraged questions |  | 0.70 |  | 0.48 |  | 0.61 | 0.63 |
| Questions were answered | 0.44 | 0.36 |  | 0.54 |  | 0.68 | 0.54 |
| Check understood infor |  | 0.58 |  | 0.53 |  | 0.63 | 0.60 |
| Language level they understood | 0.46 |  |  | 0.83 |  | 0.25 | 0.94 |
| Consent |  | 0.65 |  | 0.51 |  | 0.52 | 0.73 |
| Best care | 0.72 |  |  | 0.47 |  | 0.65 | 0.58 |
| Physical wellbeing of mother assessed |  | 0.59 |  | 0.68 |  | 0.41 | 0.83 |
| Physical wellbeing of baby assessed |  | 0.47 |  | 0.72 |  | 0.47 | 0.78 |
| Mental/emotions wellbeing assessed |  | 0.64 | 0.37 | 0.46 |  | 0.55 | 0.69 |
| Resources for emotional/mental wellbeing | | 0.50 | 0.39 | 0.56 |  | 0.51 | 0.74 |
| Other needs | 0.34 | 0.33 | 0.41 | 0.55 |  | 0.54 | 0.70 |
| Counselled |  | 0.67 |  | 0.56 |  | 0.52 | 0.73 |
| Documentation | 0.29 |  |  | 0.90 |  | 0.22 | 0.95 |
| Privacy not exposed | 0.52 |  |  | 0.61 |  | 0.50 | 0.75 |
| Auditory privacy | 0.30 |  |  | 0.72 |  | 0.50 | 0.75 |
| Information confidentiality | 0.49 |  |  | 0.68 |  | 0.54 | 0.71 |
| Pain recognition | 0.58 |  |  | 0.58 |  | 0.51 | 0.74 |
| trust | 0.73 |  |  | 0.49 |  | 0.62 | 0.61 |
| Washrooms | 0.33 |  |  | 0.76 |  | 0.39 | 0.85 |
| Cleanliness | 0.43 |  |  | 0.73 |  | 0.49 | 0.76 |
| Room temperature | 0.53 |  |  | 0.74 |  | 0.39 | 0.85 |
| Enough staff | 0.38 |  |  | 0.86 |  | 0.32 | 0.90 |
| Competence | 0.75 |  |  | 0.51 |  | 0.54 | 0.71 |
| Equipment and supplies | 0.58 |  |  | 0.72 |  | 0.38 | 0.86 |
| Safety | 0.80 |  |  | 0.43 |  | 0.61 | 0.63 |
| Vaccines | 0.62 |  |  | 0.68 |  | 0.41 | 0.83 |
| Follow up care |  | 0.33 |  | 0.76 |  | 0.47 | 0.78 |
| Disability accommodation |  |  |  | 0.99 |  |  | 1.00 |
| Wait time for provider |  |  | 0.30 | 0.89 |  |  | 0.99 |
| Wait time for labs or drugs |  | 0.34 | 0.38 | 0.75 |  |  | 1.00 |
| Time with provider |  |  |  | 0.98 |  |  | 1.00 |
| Forced into decisions |  |  |  | 0.97 |  |  | 1.00 |
| Neglected |  |  |  | 0.99 |  |  | 1.00 |
| Verbal abuse |  |  |  | 0.98 |  |  | 0.98 |
| Physical abuse |  |  |  | 1.00 |  |  | 1.00 |
| Discrimination |  |  |  | 0.99 |  |  | 1.00 |
| Blamed |  |  |  | 0.93 |  |  | 1.00 |
| Bribes |  |  |  | 0.99 |  |  | 0.99 |
| Notes: Blanks represent loading<0.2 | | | | | | | |

| **Appendix 5: Exploratory factor analysis by country of 38 retained items, CPIPE Baseline Sample for Ghana and Kenya** | | | | | | | | | | | | | | | |
| --- | --- | --- | --- | --- | --- | --- | --- | --- | --- | --- | --- | --- | --- | --- | --- |
|  | **Kenya, N=671** | | | | | | |  | **Ghana, N=705** | | | | | | |
|  | **3 factor structure** | | |  |  | **Single factor structure** | |  | **3 factor structure** | | | |  | **Single factor structure** | |
| **Subscale/items** | F1 | F2 | F3 | U |  | F1 | U |  | F1 | F2 | F3 | U |  | F1 | U |
| **Dignity and respect** |  |  |  |  |  |  |  |  |  |  |  |  |  |  |  |
| Reception | 0.54 |  |  | 0.62 |  | 0.54 | 0.71 |  | 0.52 |  |  | 0.49 |  | 0.71 | 0.50 |
| Treat you with respect | 0.60 |  |  | 0.59 |  | 0.53 | 0.72 |  | 0.41 | 0.44 |  | 0.46 |  | 0.68 | 0.54 |
| Privacy not exposed | 0.63 |  |  | 0.60 |  | 0.47 | 0.78 |  |  | 0.68 |  | 0.46 |  | 0.55 | 0.70 |
| Auditory privacy |  |  | 0.29 | 0.79 |  | 0.44 | 0.81 |  |  | 0.43 |  | 0.57 |  | 0.62 | 0.62 |
| Information confidentiality | 0.65 |  |  | 0.59 |  | 0.48 | 0.77 |  |  | 0.65 |  | 0.47 |  | 0.55 | 0.69 |
| Knowledge valued |  |  | 0.31 | 0.66 |  | 0.57 | 0.68 |  | 0.43 |  | 0.35 | 0.39 |  | 0.78 | 0.40 |
| **Communication and Autonomy** |  |  |  |  |  |  |  |  |  |  |  |  |  |  |  |
| Introductions by provider |  |  | 0.31 | 0.80 |  | 0.31 | 0.90 |  |  |  | 0.51 | 0.75 |  | 0.33 | 0.89 |
| Called preferred name |  |  | 0.52 | 0.68 |  | 0.44 | 0.81 |  | 0.56 |  |  | 0.62 |  | 0.45 | 0.80 |
| Involved in decisions | 0.37 |  | 0.47 | 0.46 |  | 0.68 | 0.54 |  | 0.68 |  |  | 0.33 |  | 0.79 | 0.38 |
| Felt Heard |  |  | 0.69 | 0.47 |  | 0.51 | 0.74 |  | 0.41 | 0.39 |  | 0.38 |  | 0.77 | 0.40 |
| Explain exams/procedures |  | 0.47 | 0.36 | 0.58 |  | 0.54 | 0.71 |  |  | 0.39 | 0.61 | 0.41 |  | 0.60 | 0.64 |
| Explain medications |  | 0.49 |  | 0.66 |  | 0.53 | 0.72 |  |  | 0.31 | 0.72 | 0.39 |  | 0.51 | 0.74 |
| Could ask any questions |  | 0.36 |  | 0.54 |  | 0.67 | 0.55 |  | 0.34 | 0.38 |  | 0.54 |  | 0.66 | 0.57 |
| Encouraged questions |  | 0.56 |  | 0.51 |  | 0.62 | 0.61 |  |  |  | 0.58 | 0.46 |  | 0.64 | 0.59 |
| Questions were answered | 0.31 | 0.42 |  | 0.48 |  | 0.72 | 0.49 |  | 0.45 |  |  | 0.55 |  | 0.67 | 0.55 |
| Check understood infor |  | 0.47 |  | 0.64 |  | 0.56 | 0.68 |  | 0.39 |  | 0.52 | 0.39 |  | 0.73 | 0.46 |
| Language level they understood | 0.22 |  |  | 0.95 |  | 0.15 | 0.98 |  | 0.42 |  |  | 0.81 |  | 0.31 | 0.91 |
| Consent |  | 0.30 | 0.48 | 0.58 |  | 0.56 | 0.68 |  |  | 0.60 |  | 0.51 |  | 0.59 | 0.65 |
| Counselled |  | 0.50 |  | 0.66 |  | 0.50 | 0.75 |  |  |  | 0.37 | 0.55 |  | 0.65 | 0.58 |
| **Responsive and Supportive care** |  |  |  |  |  |  |  |  |  |  |  |  |  |  |  |
| Wait time for provider |  |  |  | 0.96 |  | 0.18 | 0.97 |  |  |  | 0.44 | 0.80 |  | -0.02 | 1.00 |
| Wait time for labs or drugs |  |  |  | 0.96 |  | 0.04 | 1.00 |  |  |  | 0.63 | 0.58 |  | -0.02 | 1.00 |
| Best care | 0.66 |  |  | 0.52 |  | 0.59 | 0.65 |  | 0.69 |  |  | 0.39 |  | 0.73 | 0.47 |
| Safety | 0.65 |  |  | 0.57 |  | 0.52 | 0.73 |  | 0.79 |  |  | 0.37 |  | 0.70 | 0.51 |
| Physical wellbeing of mother assessed |  | 0.62 |  | 0.59 |  | 0.49 | 0.76 |  |  |  | 0.54 | 0.66 |  | 0.35 | 0.88 |
| Physical wellbeing of baby assessed |  | 0.53 |  | 0.70 |  | 0.45 | 0.80 |  |  | 0.47 |  | 0.62 |  | 0.53 | 0.72 |
| Mental/emotions wellbeing assessed |  | 0.66 |  | 0.58 |  | 0.49 | 0.76 |  |  |  | 0.73 | 0.35 |  | 0.61 | 0.63 |
| Resources for emotional/mental wellbeing |  | 0.62 |  | 0.61 |  | 0.34 | 0.88 |  |  |  | 0.73 | 0.35 |  | 0.62 | 0.61 |
| Other needs met |  | 0.42 |  | 0.70 |  | 0.49 | 0.76 |  |  |  | 0.66 | 0.43 |  | 0.58 | 0.66 |
| Companionship |  |  | 0.33 | 0.91 |  | 0.15 | 0.98 |  | 0.38 | 0.52 |  | 0.42 |  | 0.68 | 0.53 |
| Pain recognition | 0.26 |  |  | 0.80 |  | 0.45 | 0.80 |  | 0.65 |  |  | 0.53 |  | 0.60 | 0.63 |
| Trust | 0.62 |  |  | 0.62 |  | 0.49 | 0.76 |  | 0.70 |  |  | 0.36 |  | 0.75 | 0.44 |
| Washrooms |  |  | 0.23 | 0.94 |  | 0.20 | 0.96 |  | 0.31 |  | 0.40 | 0.67 |  | 0.52 | 0.73 |
| Cleanliness |  |  | 0.36 | 0.77 |  | 0.39 | 0.84 |  | 0.54 |  |  | 0.60 |  | 0.60 | 0.64 |
| Room temperature | 0.33 |  |  | 0.87 |  | 0.25 | 0.94 |  | 0.52 |  |  | 0.70 |  | 0.50 | 0.75 |
| Enough staff | 0.43 |  |  | 0.81 |  | 0.33 | 0.89 |  | 0.39 |  |  | 0.84 |  | 0.31 | 0.90 |
| Competence | 0.54 |  |  | 0.71 |  | 0.43 | 0.82 |  | 0.79 |  |  | 0.41 |  | 0.65 | 0.58 |
| Vaccines | 0.35 |  |  | 0.89 |  | 0.25 | 0.94 |  | 0.62 |  |  | 0.62 |  | 0.52 | 0.73 |
| Follow up care |  | 0.38 |  | 0.74 |  | 0.38 | 0.85 |  |  | 0.58 |  | 0.55 |  | 0.57 | 0.67 |
| Notes: F=Factor Loading; U= Uniqueness. Blanks represent loadings <0.2 for 3 factor structure | | | | | | | | | | | | | | | |
